# Knowledge, Attitude and Practice during the COVID-19 Pandemic in South-East Gabon

**DOI:** 10.3390/bs12070226

**Published:** 2022-07-11

**Authors:** Roméo Karl Imboumy-Limoukou, Barthélemy Ngoubangoye, Serge Ely Dibakou, Sandrine Lydie Oyegue-Liabagui, Franck Mounioko, Lady Charlene Kouna, Walter Roddy Matangoye, Steede Seinnat Ontoua, Nancy Cheronne Mbani Mpega, Jean-Bernard Lekana-Douki

**Affiliations:** 1 Centre International de Recherches Médicales de Franceville, BP 769 Franceville, Gabon; sergeelydibakou@cirmf-gabon.com (S.E.D.); sandrineoyegue@cirmf-gabon.com (S.L.O.-L.); franckmounioko@cirmf-gabon.com (F.M.); charleneklc@gmail.com (L.C.K.); matangoyew@gmail.com (W.R.M.); ontouaseinnat@gmail.com (S.S.O.); fayardmouyopa@gmail.com (N.C.M.M.); jb.lekanadouki@cirmf-gabon.com (J.-B.L.-D.); 2 Laboratoire de Recherches en Immunologie, Parasitologie et Microbiologie, Ecole Doctorale Régionale d’Afrique Centrale en Infectiologie Tropicale (ECODRAC), Université des Sciences et Techniques de Masuku, BP 876 Franceville, Gabon; 3 Département de Parasitologie-Mycologie, Université des Sciences de la Santé, BP 4009 Libreville, Gabon

**Keywords:** COVID-19, Gabon, knowledge, practice, perceptions, attitude, rural, urban

## Abstract

COVID-19 is an emerging respiratory disease; it was declared a global pandemic by the World Health Organization. This survey aimed to describe the knowledge, attitudes, perception and practices of the Gabonese public towards COVID-19. This study was performed on 1016 participants. All participants filled in the questionnaire voluntarily, reporting demographic characteristics and answering questions assessing their level of knowledge, attitudes, perceptions and practice towards COVID-19. Among participants, there were 535 men and 476 women. The mean age of the participants was 33.2 ± 16.7 years old. Almost all participants (98.1%) said that they had heard about COVID-19 but only 2.8% knew the pathogenic agent responsible for COVID-19. More than 80% knew that the disease could be transmitted by greeting infected people (87.3%), kissing an infected person (90.0%), touching an infected doorknob (83.5%) and attending meetings (83.9%). The mean knowledge score was higher among younger than older participants, higher among participants living in urban areas than those living in rural areas and higher among participants with higher levels of education than those with lower levels of education. In general, respondents had good knowledge of COVID-19 and a positive attitude towards using protective measures; however, there were differences according to gender, age group, place of residence, professional group and level of education.

## 1. Introduction

In December 2019, coronavirus disease 2019 (COVID-19) emerged in Wuhan, China. COVID-19 is a highly contagious disease which has spread to more than 200 countries and has been declared a global pandemic by the World Health Organization (WHO) [1,2].

To date (January 2021), it has infected nearly 86 million people, of whom 2,455,131 have died as a result of the disease [3]. COVID-19 is caused by SARS-CoV-2 (Severe Acute Respiratory Syndrome Coronavirus 2), an enveloped virus with positive single-stranded RNA belonging to the family Coronaviridae. Members of this family cause flu-like symptoms which can range from a simple cold to severe symptoms, such as severe acute respiratory syndrome (SARS) [4,5]. This is the case of SARS-CoV-1 (Severe Acute Respiratory Syndrome Coronavirus 1), causing more than 8000 cases with 10% mortality and MERS-CoV (Middle East Respiratory Syndrome Coronavirus) causing 857 cases with 35% mortality [6]. 

There are now several vaccines with varying protection rates but there are none available in Gabon and no definitive therapy for SARS-CoV-2 has been developed. The application of preventive measures remains the best means of fighting SARS-CoV-2, especially in Africa where many health systems are not well equipped to face this epidemic. 

Preventive measures have been promoted by the World Health Organization (WHO) and relayed by the health authorities to curb the spread of the virus. Compliance with these control measures is essential and is heavily influenced by the knowledge, attitudes and practices (KAP) towards COVID-19 [7,8].

In Gabon, the first case of COVID-19 was declared on 12 March 2020, involving a Gabonese person returning from holidays in France. Since this case was declared, 14,234 cases have been confirmed, with more than 70 deaths (24 February 2021), despite the awareness campaigns implemented by the Gabonese government via the media. “Fake news” propagated on social networks and relayed by word of mouth creates panic in the population and reduces compliance with the awareness messages communicated by authorities [9,10]. In the media, awareness messages are generally in French, whereas some people in rural areas speak only their local language. This situation could impede the public health response. To adapt public health response strategies, studies of knowledge, attitudes and practices can provide baseline data to determine the type of intervention that may be required to change misconceptions about SARS-CoV-2 in the community. 

In this context, the objective of this study is to understand the knowledge, perceptions, attitudes and practices relating to COVID-19 among people living in rural and urban areas in Gabon. Since the pandemic was declared, no such studies have been conducted in Gabon.

## 2. Materials and Methods

### 2.1. Study Area

The study was conducted in rural and urban areas of southeast Gabon. The urban area was Franceville, the capital of the Haut-Ogooué Province, and the third largest town in Gabon in terms of population (pop. 110,568 inhabitants) [11]. The rural areas were 9 villages in Lombo-Bouenguidi, department of Ogooué-lolo Province: Toundi-Odounga, Dienga, Iwetsi, Grand Village, Mourombo-Fouala, Maranda1, Maranda2, Siono and Isseme. These villages are situated near the border with the Republic of Congo and have in total around 2000 inhabitants.

### 2.2. Study Design and Population

This community-based, cross-sectional, descriptive study was conducted from 7 to 11 September 2020. The study population was composed of inhabitants aged 10 years old and older. Inclusion criteria stipulated that participants must reside in the study site during the study. It is crucial to note that one possible problem with surveys that ask respondents for either their policy support for or compliance with COVID-19 policies is the presence of social desirability bias, according to [12].

### 2.3. Sampling

In villages, all the people concerned (aged 10 years old and older) were interviewed. In Franceville, we divided the city into eight (8) points. 

From these points, investigations were carried out simultaneously in a random manner. To reduce bias of household selection, the first house visited was determined by a random method. We assigned a number (1–8) to each interviewer and let the interviewer select the first house on the basis of counting that number from the closest house to the drop-off point. The sampling interval was then used to select subsequent households. The sampling interval was 10 (i.e., every 10th house would be visited).

### 2.4. Study Tools

The survey questionnaire was designed in French, the national language in Gabon, and translated into local languages (*Nzebi, Obamba and Téké* languages) by the interviewers when necessary. The surveys started with a short presentation of the project, and we asked participants to say whether they agreed to participate in the study voluntarily. We conducted in person interviews, approximately 30 min in length. These interviews were used to explain the objectives of the project as misinformation has created mistrust and not everyone has the same level of understanding. Questions covered socio-demographic characteristics of the participant, knowledge of COVID-19, attitudes to protective measures against COVID-19 and practices relevant to COVID-19. In order to determine the level of knowledge of the participants, we asked questions on the general knowledge of COVID-19 with a questionnaire including predefined answers, in particular on the symptoms, the means of transmission, the prevention measures and the perception of the disease. Answers were Yes, No or I don’t know. 

### 2.5. Statistical Analysis

We assessed each participant’s knowledge of COVID-19 as described previously [13]. We gave one point for all correct responses and zero points for unanswered questions or incorrect answers. 

We conducted Student’s *t* and ANOVA tests using R software Version 4.0.2 to test the relationship between knowledge score and socio-demographic variables. Where ANOVA tests were significant, we used the Tukey correction for multiple comparisons.

All *p* values were based on two-sided tests and were considered statistically significant at *p* < 0.05.

### 2.6. Ethical Considerations

The Gabonese National Ethics Committee (CNER) approved our study protocol and procedures, (N°003/2020/CNER/SG/P of 1 February 2020). Respondents’ anonymity and confidentiality were ensured by assigning numbers to participants. “Informed consent was obtained from all subjects involved in the study”. Furthermore, “Written informed consent has been obtained from all the patients to publish this paper”. For children, consent was sought from their parents/guardians, as recommended by CNER.

## 3. Results

A total of 1016 participants completed the survey. The mean age was 33.2 ± 16.7; 535 (52.7%) were male and 476 (46.9%) were female. Of the 1016 respondents, 494 (48.6%) resided in rural areas (out of a total of 2000 people) whereas 522 (51.4%) resided in urban areas (out of a population of 5000 people).

Nearly two thirds (63.1%) were single. More than half (59.1%) had completed higher studies, 24.0% had preparatory or high school education, 11.3% were university graduates and 5.0% had no education. About one third (35.3%) of participants were students. Other demographic characteristics are detailed in Table 1.

Almost all participants (98.1%) said that they had heard about COVID-19 and 8.3% of them believed that the disease does not exist. Only 2.8% knew the pathogenic agent of COVID-19. 

More than 80% knew that the disease could be transmitted by greetings (87.3%), kissing an infected person (90.0%), touching infected doorknobs (83.5%) and attending meetings (83.9%).

Regarding the symptoms of COVID-19, the respondent’s answers were: 85.1% fever, 78.6% headaches, 55.5% aches and pains, 99.0% dry cough, 32.8% stomach ache, 70.3% loss of taste, 51.3% vomiting, 83.9% difficulty breathing, 74.0% throat irritation, 74.6% fatigue and 75.7% runny nose (Table 2). In terms of prevention measures, almost all participants knew that washing hands (94.5%), wearing a mask (94.7%) and maintaining an appropriate distance (1 m) between yourself and anyone with symptoms (93.0%) were the best ways to prevent COVID-19. Furthermore, 76.0% thought that avoiding touching a contaminated surface and then touching your eyes could prevent COVID-19 infection. More than half of respondents thought that the disease could be prevented using steam baths (50.6%) and 44.1% of participants thought that traditional medicines could be effective against the disease. For further details, see Table 2. 

Concerning participant’s perceptions of and attitudes to COVID-19, 91.9% of participants thought that it is dangerous, 90.7% thought it could kill, 46.3% thought it is a laboratory invention and 63.3% thought that it affects only white people. Of people interviewed, 29.1% still continued to greet people with a handshake, 90.1% said they wore a mask in public places, 88.1% said they washed their hands regularly and 62.2% of respondents stated that they were following the global pandemic situation. Furthermore, 77.0% accepted the quarantine measures, 52.0% were in favour of vaccination but only 20.9% thought vaccination would protect them. In addition, 81.3% of participants said they would wear protective and control measures to limit the spread of the disease. Further aspects of participant perceptions and attitudes are reported in Table 3.

Knowledge scores were significantly different across gender, age group, area of residence, occupation group and level of education (Table 4). Men’s knowledge score was higher than women’s (*p* < 0.001). In general, age group had a significant impact on knowledge scores (*p* < 0.001). The age groups under 40 have significantly higher knowledge scores than the age groups over 40. There are significant differences between 10–20 and 40–50 (*p* = 0.003), between 10–20 and over 60 years (*p* < 0.001), between 20–30 and 40–50 (*p* = 0.001), between 20–30 and over 60 years old (*p* < 0.001) and between 30–40 and over 60 years old (*p* < 0.001) (Table 4). Respondents living in urban areas had a significantly higher knowledge score than rural residents (*p* < 0.001). Participants with university education or higher had significantly higher knowledge scores than those with lower levels of education. Employed participants had higher scores than unemployed participants. 

The comparison of knowledge scores by questionnaire item shows that the urban population is better informed about COVID-19 symptoms and preventive measures than the rural population (*p* < 0.001) (Table 4). 

## 4. Discussion

Even if Africa has been relatively less affected than the other continents [14,15], the disease continues to spread and remains the primary health concern of the continent. In Gabon, various awareness programs have been put in place (television, radio, social networks and internet) by the health authorities and measures including a ban on grouping, curfews and mask-wearing have been put in place to prevent the disease. However, no study has yet been carried out on the level of knowledge and good practices towards COVID-19. We present and discuss here the results of a survey of the knowledge, perceptions and attitudes of people in south-eastern Gabon towards the disease. 

### 4.1. Knowledge of Means of Transmission, Symptoms and Prevention Measures

The surveys show that people we interviewed had a good general knowledge of COVID-19. Almost the entire sample had heard of COVID-19 and the majority were aware of the prevention measures, mode of transmission and most of the symptoms. This good general knowledge is consistent with other studies in the world [13,16,17,18] and may be due to efforts by the government via awareness campaigns and daily warning messages sent from the government and the Ministry of Health to everyone’s mobile telephones and awareness campaigns on national media channels. The result may also be explained by the relatively young age of the majority of the study population and their frequent use of social media platforms, knowing that in 2020 the internet represented the most important information source [19]. However, (i) less than 3% of respondents knew the pathogenic agent and those who did were from urban areas and had a high level of education. This may be because awareness campaigns are focused on prevention and people usually called the disease coronavirus rather than COVID-19, so they did not know that SARS-CoV-2 is the virus responsible for COVID-19. (ii) 32.0% of respondents thought that antibiotics could be effective against COVID-19 and 23.4% did not know if antibiotics could cure COVID-19. The purchase of non-prescribed antibiotics is a common practice in Sub-Saharan Africa [19], and the panic caused by the COVID-19 pandemic [9] may lead to the consumption of antibiotics, which is known to be a major driver of antimicrobial resistance [19]. (iii) Only 20.9% of the sample think that the vaccine can stop the disease and 46.26% think that the vaccine will not protect against the disease. This result could be explained by the misinformation that has circulated on social networks about vaccines that are harmful to the reproduction of the African population and that vaccination would allow the control of populations. Specific immunization awareness programs should be conducted by the authorities to address this. (iv) Urban populations have better knowledge of COVID-19, symptoms and means of prevention. These results could be explained by the limited access to the media in rural areas, whereas in urban areas there are diverse sources of information. Women have lower scores than men and educated people have better levels of knowledge than uneducated people. However, these differences are very small, although significant. As envisaged by Zhang et al., specific outreach programs could be designed to target particular demographic social groups [20], such as villagers, women or uneducated or poorly educated populations. 

### 4.2. Perceptions of Participants 

Almost all (91.9%) of the respondents believe that COVID-19 threatens life and may be a cause of death (90.7%). These results show that populations have understood the dangerousness of the disease. However, nearly half (49.4%) think the effects of the disease are exaggerated. These perceptions may be due to media pressure. At the beginning of the pandemic, the media covered it intensely. Almost half (46.3%) of the people interviewed thought that COVID-19 was manufactured in a lab. This proportion is much higher than the proportion found by [21]; this could be explained by the particular African continent context and especially the timing of the study.

More than half thought COVID-19 is a disease of “white people” and that a hot climate could stop the spread of the disease. This pattern may be because people’s major source of information is social media. Fake news, misinformation and rumour-mongering are common due to a surge in the use of the internet and social media [13,22,23]. Unfortunately, information from social networks seems to circulate more easily in the general population than the scientific information which rejects this hypothesis [24].

### 4.3. Attitudes of Participants towards Control Measures

The majority of respondents took the precautions necessary to avoid contracting COVID-19. More than three-quarters of participants said they wear a mask in public places, wash their hands regularly, respect protective and control measures, will accept quarantine if they test positive and will contact the health authorities if they test positive. More than half say that they are informed about the progress of the pandemic. This positive attitude may result from information about the disease being regularly updated on various mass media channels and the government ensuring that its measures are enforced by the presence of military personnel in public places. 

More than half of the study population was willing to obtain the vaccine once it was available, consistent with a study in Egypt [13]. These results show people’s confidence in vaccines, but this result contrasts with vaccine perception because only 20.9% of respondents think that vaccination can protect them. This contradiction illustrates the ambiguity around the COVID-19 vaccine. This ambiguity is illustrated by nearly 47 percent of the population not being ready to be vaccinated if the vaccine is available. This reluctance, already expressed in other studies in Israel or Pakistan [25,26], could be a barrier and a substantial challenge in the fight against the pandemic. 

The fight against the pandemic must also include the fight against false information and conspiracy theories circulating on social networks on the quality of vaccines. Some examples are that vaccines would represent a health risk because they have been developed rapidly, the presence of active virus in the vaccines or nano-chips imbedded to control the population with the 5G towers telephone network [26]. 

## 5. Conclusions

This study is the first to examine the knowledge, attitudes and practices of Gabonese people relating to COVID-19. In general, respondents had good knowledge of COVID-19 and a positive attitude towards using protective measures, but there were differences according to gender, age group, place of residence, professional group and level of education.

## Figures and Tables

**Table 1:** Socio-demographic characteristics of the participants (*n* = 1016).

Socio-Demographic Characteristics	Number (N°)	Percentage (%)
**Sexes**		
Male	535	52.66
Female	476	46.85
**Age groups in years**		
10–20	223	21.95
20–30	293	28.84
30–40	198	19.49
40–50	142	13.98
50–60	80	7.87
≥60	79	7.78
**Area of residence**		
Urban	494	48.62
Rural	522	51.38
**Profession**		
Student	359	35.33
Retired	15	1.48
Unemployed	304	29.92
Employed	337	33.17
**Level of education**		
Primary school	244	24.02
None	51	5.02
High school	600	59.06
University	115	11.32
**Marital status**		
Single	641	63.09
Cohabitation	205	20.18
Married	123	12.11
Widower	47	4.63

**Table 2:** Knowledge about COVID-19 among the participants (*n* = 1016).

Knowledge Items	Yes		No		Don’t Know	
	No	%	No	%	No	%
**COVID-19**						
Have you ever heard of COVID-19?	997	98.13	11	1.08	8	0.79
Does it exist?	803	79.04	84	8.27	129	12.70
Pathogenic agent?	28	2.76	5	0.49	983	96.75
**Route of transmission (how the disease is spread)**						
By salt water?	186	18.31	629	61.91	201	19.78
By greetings?	887	87.30	86	8.46	43	4.23
By kissing people?	914	89.96	52	5.12	50	4.92
By sexual relationships?	461	45.37	346	34.06	209	20.57
By goods from China?	494	48.62	285	28.05	237	23.33
By eating bushmeat?	260	25.59	577	56.79	179	17.62
By touching door cuff?	848	83.46	70	6.89	98	9.65
By being in contact with stool/urine?	311	30.61	475	46.75	230	22.64
By attending meetings?	852	83.86	74	7.28	90	8.86
By being in closed space?	293	28.84	598	58.86	125	12.30
By playing with animals (pets)?	338	33.27	467	45.96	211	20.77
By being in contact with asymptomatic people?	464	45.67	383	37.70	169	16.63
**Symptoms (What are the symptoms of COVID-19)**						
Fever?	865	85.14	81	7.97	70	6.89
Headaches?	799	78.64	133	13.09	84	8.27
Soreness?	564	55.51	320	31.50	132	12.99
Dry cough?	833	81.99	98	9.65	85	8.37
Stomach aches?	333	32.78	505	49.70	177	17.42
Lost of taste?	714	70.28	181	17.81	121	11.91
Vomiting?	521	51.28	332	32.68	163	16.04
Difficulty breathing?	852	83.86	74	7.28	90	8.86
Sore throat?	752	74.02	153	15.06	111	10.93
Tiredness?	758	74.61	152	14.96	106	10.43
Runny nose?	769	75.69	105	10.33	142	13.98
**Prevention measures to avoid COVID-19** **(What are the preventive measures to avoid COVID-19?)**						
Wash hands?	960	94.49	17	1.67	39	3.84
Wear a mask?	962	94.69	21	2.07	33	3.25
Maintain a distance of 1 m from other people?	945	93.01	44	4.33	26	2.56
Take antibiotics?	325	31.99	453	44.59	238	23.43
Take traditional medicines?	448	44.09	393	38.68	174	17.13
Do steam baths?	514	50.59	342	33.66	160	15.75
Avoid touching nose/eyes?	772	75.98	134	13.19	110	10.83
Drink garlic, ginger, lemon infusions?	452	44.49	335	32.97	229	22.54
Is there a vaccine against COVID-19?	212	20.87	470	46.26	334	32.87
Is there a treatment?	505	49.70	271	26.67	239	23.52

**Table 3:** Perceptions and attitudes of the participants about COVID-19 (*n* = 1016).

Items	Yes		No		Don’t Know	
	No	%	No	%	No	%
**Participants’ perception of COVID-19**						
Is COVID-19 a dangerous disease?	934	91.93	35	3.44	47	4.63
Is it a fatal disease?	921	90.65	45	4.43	50	4.92
A shameful disease?	312	30.71	609	59.94	95	9.35
Is it a stigmatizing disease?	547	53.84	332	32.68	137	13.48
Do you think we are exaggerating about this disease?	502	49.41	380	37.40	133	13.09
Do you think the virus was made in the laboratory?	470	46.26	265	26.08	279	27.46
Can a person have COVID-19 multiple times?	436	42.91	306	30.12	274	26.97
Is it a disease that kills white people more?	643	63.29	246	24.21	127	12.50
Do cold climates favour the disease?	650	63.98	172	16.93	194	19.09
Do warm climates slow the spread?	636	62.60	204	20.08	176	17.32
**Attitude of participants to COVID-19**						
Among colleagues, do you greet each other by shaking hands?	296	29.13	7	0.69	713	70.18
Do you wear a mask in public spaces?	917	90.26	92	9.06	7	0.69
Do you wash your hands regularly?	895	88.09	116	11.42	5	0.49
Do you follow the news about the pandemic?	632	62.20	379	37.30	5	0.49
Will you agree to quarantine yourself if you test positive?	782	76.97	229	22.54	5	0.49
Has wearing a mask become a reflex for you?	676	66.54	336	33.07	4	0.39
If you are in contact with a positive person, will you contact the health authorities?	840	82.68	172	16.93	4	0.39
If a vaccine is available, will you get vaccinated?	528	51.97	483	47.54	5	0.49
Do you still respect the barrier measures?	826	81.30	185	18.21	5	0.49

**Table 4:** Relationship between socio-demographic characteristics of the participants and their knowledge scores about COVID-19 (*n* = 1016).

Socio-Demographic Characters	Category	Knowledge Score		Test of sig. (*p*)
		Min-Max	Mean SD	
**Sex**				t = −4.14 (<0.001) *
Male		0–33	21.1 ± 5.0	
Female		0–33	20.0 ± 5.0	
**Age (years)**				F = 10.57 (<0.001) *
10–20	a	0–31	21.4 ± 4.1	e vs. f = 0.05, c vs. f < 0.001 ^#^
20–30	b	1–33	21.4 ± 4.1	b vs. f < 0.001 ^#^, a vs. f < 0.001 ^#^
30–40	c	0–33	20.3 ± 5.6	b vs. d = 0.001 ^#^, a vs. d = 0.003 ^#^
40–50	d	0–28	19.4 ± 6.0	
50–60	e	1–27	20.0 ± 5.0	
≥60	f	0–28	17.6 ± 6.0	
**Are of residence**				t = −5.94 (<0.001) *
Urban		2–33	21.4 ± 4.4	
Rural		0–29	19.6 ± 5.4	
**Profession**				F = 15.56 (<0.001) *
Student	a	1–33	22.0 ± 4.0	d vs. c < 0.001 ^#^, a vs. c < 0.001 ^#^
Retired	b	17–28	21.0 ± 3.0	a vs. d = 0.001 ^#^
Unemployed	c	0–28	19.0 ± 6.0	
Employed	d	0–33	20.4 ± 5.0	
**Level of education**				F = 40.2 (<<0.001) *
Primary school	a	0–29	19.4 ± 5.0	a vs. b < 0.001 ^#^, c vs. b < 0.001 ^#^
None	b	0–27	15.0 ± 8.4	d vs. b < 0.001 ^#^, c vs. a < 0.001 ^#^
High school	c	0–31	24.0 ± 4.3	d vs. a < 0.001 ^#^, d vs. c < 0.001 ^#^
University	d	14–33	23.1 ± 4.0	
**Marital status**				F = 12.24 (<0.001) *
Cohabitation	a	0–28	19.4 ± 6.1	b vs. d < 0.001 ^#^, b vs. d < 0.001 ^#^
Single	b	0–33	21.2 ± 4.4	b vs. c = 0.003 ^#^
Married	c	1–28	19.5 ± 5.0	
Widower	d	0–26	18.4 ± 6.0	

t Student’s *t* test, **F** analysis of variance (ANOVA) test. * Statistically significant at *p* < 0.05. ^#^ Categories of variables with significant ANOVA results. Multiple comparisons between each 2 categories are done by post hoc analysis (Tukey).

## Data Availability

Not applicable here.
